# Supplementary material for: Intermanual transfer of visuomotor adaptation is related to awareness
Source: PLoS One. 2019 Sep 6;14(9):e0220748. doi: 10.1371/journal.pone.0220748 (PMC6730885; doi:10.1371/journal.pone.0220748)
Supplement: S1 Model — (PDF) [file pone.0220748.s001.pdf]

Model of Bayesian analysis

We fit a regression model to the mean movement directions of each subject in each episode. The regression model has a term for the episode type, subject group, and subject. It also includes terms for the interaction of episode and group and episode and subject. All the coefficients of the regression model are assumed to be normally distributed with a broad, uninformative prior. Noise around the regression model is assumed to have a normal distribution with a standard deviation that varied between subjects but was constant within subject. The per-subject standard deviation has a gamma distribution whose parameters are sampled hierarchically and has broad priors.

```
model{

# N observations (subjects * withinX1 * betweenX2 in my case)
for(i in 1:N){
    y[i] ~ dnorm(muY[i], tauY[id[i]])
    muY[i] <- a0 + a1[X1[i]] + a2[X2[i]] + a12[X1[i],X2[i]] + aS[id[i]] + a1S[X1[i],id[i]]
}

# Priors

a0 ~ dnorm(0, a0Tau)
for (j1 in 1:nX1) {
    a1[j1] ~ dnorm(0, a1Tau)
}

for (j2 in 1:nX2) {
    a2[j2] ~ dnorm(0, a2Tau)
}
for (j1 in 1:nX1) {
    for (j2 in 1:nX2) {
        a12[j1,j2] ~ dnorm(0, a12Tau)
    }
}

for(i in 1:nS){
    aS[i] ~ dnorm(0, tauS)
    tauY[i] ~ dgamma(tauYRa, tauYSh)
}

for (j1 in 1:nX1) {
    for (s in 1:nS) {
        a1S[j1,s] ~ dnorm(0, tauA1S)
    }
}

tauYRa <- (tauYMode + sqrt(tauYMode^2 + 4*tauYVar))/(2*tauYVar)
tauYSh <- 1 + tauYMode*tauYRa

tauYMode ~ dgamma(tauYModeSh, tauYModeRa)
tauYVar ~ dgamma(tauYVarSh, tauYVarRa)
```
