# Supplementary material for: Intermanual transfer of visuomotor adaptation is related to awareness
Source: PLoS One. 2019 Sep 6;14(9):e0220748. doi: 10.1371/journal.pone.0220748 (PMC6730885; doi:10.1371/journal.pone.0220748)
Supplement: S1 Statistics — (PDF) [file pone.0220748.s002.pdf]

Supplementary statistical analysis

For additional statistical analyses we performed several analyses of variances (ANOVAs). We used mixed model ANOVAs on the Adaptation indices with Episode as a within-subject factor and Group as a between-subject factor. One way ANOVAs were used for the awareness, unawareness, transfer and washout indices with Group as the factor. Normality within each index for each group separately was explored by Kolmogorov-Smirnov-Test and variance stability across the factor Group was explored by Levene´s test. Greenhouse-Geisser-adjustments were applied when necessary to compensate for heterogeneity of variances. Main effects were explored with LSD post-hoc tests. In addition, partial correlations between awareness and transfer indices as well as between awareness and normalized transfer indices with the control variable Group were calculated. All statistical comparisons were performed using SPSS (Version 25.0. Armonk, NY: IBM Corp.). Probability of type I error (*p*) is reported as a number unless it is less than 0.001. Effects sizes are reported using Cohen's *d* for t tests and the explained variation η^2 for ANOVAs.

Adaptation

Results from ANOVA:

- main effects of Group ($p < 0.001$, $\eta^2 = 0.52$), Episode ($p < 0.001$, $\eta^2 = 0.64$) and Group × Episode ($p < 0.001$, $\eta^2 = 0.22$)
- post-hoc analysis reveals a difference between the first and last episode within each group (G30 ($p < 0.001$, $d = 4.49$), S30 ($p < 0.001$, $d = 4.57$), G75 ($p < 0.001$, $d = 2.80$), S75 ($p < 0.001$, $d = 3.18$))
- for the last adaptation episode post-hoc analysis shows a smaller adaptation index for G75 than all other groups (G30 ($p = 0.012$, $d = 1.16$), S30 ($p = 0.037$, $d = 1.13$), S75 ($p < 0.001$, $d = 1.23$))
- within episodes 10 to 25 post-hoc analysis reveals no differences for groups G30, S30 and S75 (all 45 comparisons p > 0.05; episode 20 for example: G30/S30 (p = 0.890), G30/S75 (p = 0.331), S30/S75 (p = 0.403))

⇨ Statistical analyses reveal that adaptation took place in all groups and that group G75 adapted less than the other three groups.

Awareness, unawareness, intermanual transfer and washout

Results from one-way ANOVAs:

- Awareness: main effect of Group ($p < 0.001$, $\eta^2 = 0.35$) with awareness index of S75 being larger than that of all other groups (G30 ($p < 0.001$, $d = 1.56$), S30 ($p < 0.001$, $d = 1.46$), G75 ($p = 0.009$, $d = 0.75$))
- Unawareness: main effect of Group ($p < 0.001$, $\eta^2 = 0.57$) with unawareness index of S75 being smaller than that of all other groups (G30 ($p < 0.001$, $d = 2.58$), S30 ($p < 0.001$, $d = 2.58$), G75 ($p < 0.001$, $d = 1.24$)) and unawareness index of G75 being smaller than that of G30 ($p = 0.011$, $d = 1.18$) and S30 ($p = 0.026$, $d = 1.06$)
- Intermanual transfer: main effect of Group ($p = 0.004$, $\eta^2 = 0.26$) with transfer index of S75 being larger than that of all other groups (G30 ($p < 0.001$, $d = 1.24$), S30 ($p = 0.021$, $d = 0.77$), G75 ($p = 0.004$, $d = 0.81$)
- Normalized intermanual transfer: main effect of Group ($p = 0.028$, $\eta^2 = 0.19$) with transfer index of S75 being larger than G30 ($p = 0.003$, $d = 1.12$)
- Washout: main effect of Group ($p < 0.001$, $\eta^2 = 0.58$) with washout index of S75 being smaller than that of all other groups (G30 ($p < 0.001$, $d = 2.80$), S30 ($p < 0.001$, $d = 2.31$), G75 ($p < 0.001$, $d = 1.18$)) and washout index of G75 being smaller than that of G30 ($p < 0.001$, $d = 1.46$) and S30 ($p < 0.001$, $d = 1.29$)

⇨ Statistical analyses reveal the same pattern of results for awareness and transfer indices with S75 being larger than all other groups (larger than G30 for normalized transfer). Similarly, the analyses of unawareness and washout indices both yield differences of S75 and G75 to all other groups.

Results of correlations:

- correlation of awareness and transfer with $R = 0.65$ ($p<0.001$) and of awareness and normalized transfer with $R = 0.70$ ($p<0.001$)

⇨ Statistical analyses reveals that the size of awareness indices correlates with intermanual transfer even when transfer is expressed in relation to final adaptation level.
