# Supplementary material for: Intermanual transfer of visuomotor adaptation is related to awareness
Source: PLoS One. 2019 Sep 6;14(9):e0220748. doi: 10.1371/journal.pone.0220748 (PMC6730885; doi:10.1371/journal.pone.0220748)
Supplement: S2 Results — (PDF) [file pone.0220748.s006.pdf]

Movement paths of the first adaptation episode of individual subjects

Movement paths in S75 are more irregular than in the other groups.

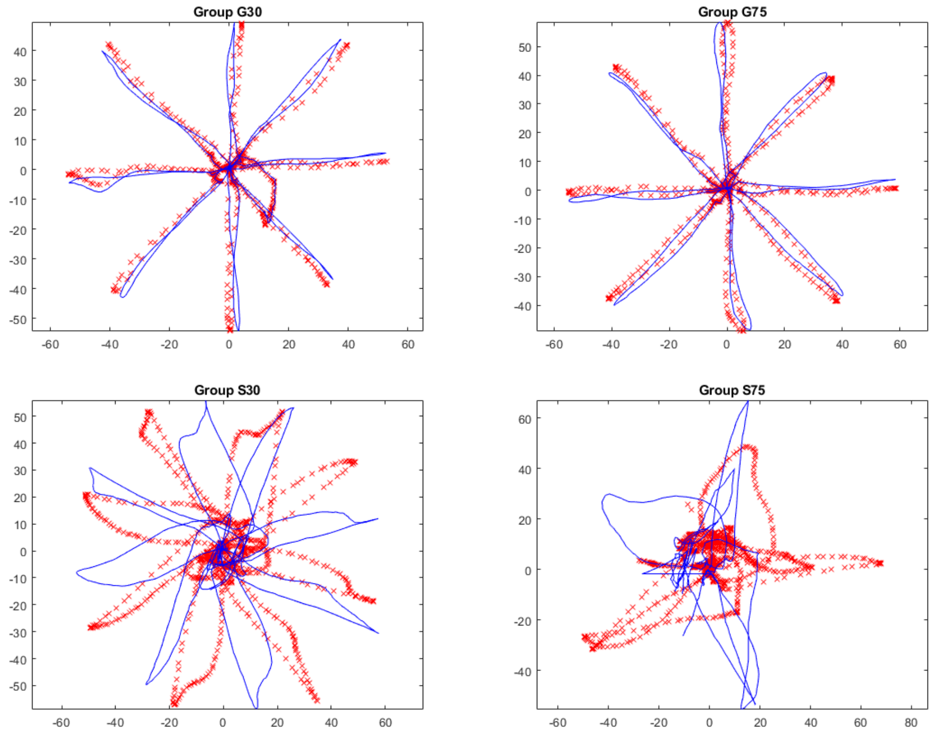

Fig. 1: Examples of movement paths. We show original registrations of movement paths (red crosses) and cursor paths (blue lines) of the first adaptation episode produced by a typical participant of each group.
