# Supplementary material for: Intermanual transfer of visuomotor adaptation is related to awareness
Source: PLoS One. 2019 Sep 6;14(9):e0220748. doi: 10.1371/journal.pone.0220748 (PMC6730885; doi:10.1371/journal.pone.0220748)
Supplement: S3 Results — (PDF) [file pone.0220748.s007.pdf]

Results of the analysis of the refresh phase

Learning continued during refresh phase for group G75.

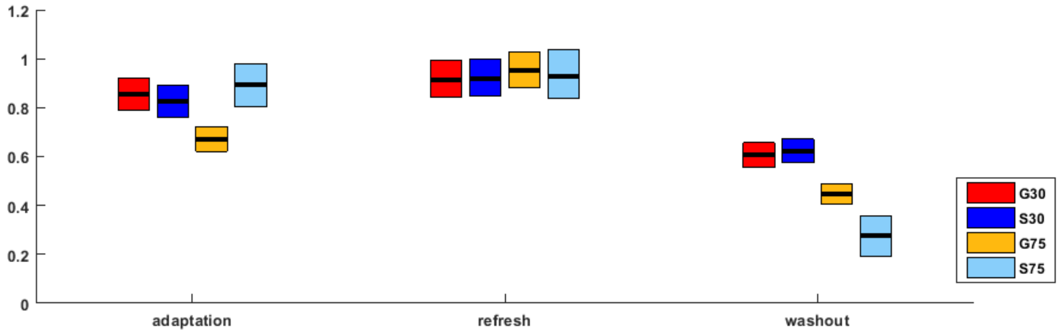

Fig. 1: Adaptation, refresh and washout. Group means and their HDIs of posterior probability distributions for the last episode of adaptation, all six refresh episodes and the first washout episode.

	**Adaptation**	**Refresh**	**Washout**
	S75	S75	S75
G30	43 %	56 %	< **0.1 %**
S30	8 %	56 %	< **0.1 %**
G75	8 %	54 %	< **0.1 %**
	G75	G75	G75
G30	19 %	55 %	7 %
S30	64 %	57%	18 %
	S30	S30	S30
G30	19 %	65 %	67 %

Tab. 1: Results of between group analysis for adaptation, refresh and washout. Effective equality of posterior distribution of the differences between the estimates of the four group indices of adaptation, refresh and washout indices using a ROPE of -0.05 to 0.05. Significant differences are marked in bold numbers.

	**G30**	**S30**	**G75**	**S75**
	adaptation	adaptation	adaptation	adaptation
refresh	39 %	25 %	**< 0.1 %**	39 %
washout	**< 0.1 %**	**< 0.1 %**	**< 0.1 %**	**< 0.1 %**
	refresh	refresh	refresh	refresh
washout	**< 0.1 %**	**< 0.1 %**	**< 0.1 %**	**< 0.1 %**

Tab. 2: Tab. 1: Results of within group analysis for adaptation, refresh and washout. Effective equality of posterior distribution of the differences between the estimates of the adaptation, refresh and washout indices within each group using a ROPE of -0.05 to 0.05. Significant differences are marked in bold numbers.
